# Unveiling the Frontiers of Synthetic Biology in Brazil: Pioneering the National Synthetic Biology Network

**DOI:** 10.1021/acsomega.5c03077

**Published:** 2025-07-23

**Authors:** Leandro V. Santos, Giovanna R. Maklouf, Cibele Z. S. do Nascimento, Milca R. C. R. Lins, Juliana C. Rezende, Cristian A. Rojas, Yala Sampaio, Nájua K. El Kadri, Guilherme E. Kundlatsch, Rita C. G. Simão, Elibio L. Rech, Luiziana F. Silva, Danielle B. Pedrolli

**Affiliations:** † Genetics and Molecular Biology Graduate Program, Institute of Biology, 28132 State University of Campinas (Unicamp), Campinas, São Paulo 13083-862, Brazil; ‡ Manchester Institute of Biotechnology, University of Manchester, Manchester M1 7DN, U.K.; § Brazilian Association of Synthetic Biology (SynBioBR), São Paulo, São Paulo 01310-200, Brazil; ∥ Center for Natural and Human Sciences, Federal University of ABC (UFABC), Santo André, São Paulo 09210-580, Brazil; ⊥ Latin American Institute of Life and Nature Sciences, 245075 Federal University for Latin American Integration, Foz do Iguaçu, Paraná 85870-650, Brazil; # Institute of Biological Sciences, 28114 Federal University of Minas Gerais (UFMG), Belo Horizonte, Minas Gerais 31270-901, Brazil; ¶ Department of Bioprocess Engineering and Biotechnology, Federal University of Paraná (UFPR), Curitiba, Paraná 81531-990, Brazil; ∇ School of Pharmaceutical Sciences, Department of Bioprocess Engineering and Biotechnology, São Paulo State University (Unesp), Araraquara, São Paulo 14800-903, Brazil; ○ Center of Medical and Pharmaceutical Sciences, Western Paraná State University (Unioeste), Cascavel, Paraná 85819-110, Brazil; ⧫ National Institute of Science and Technology—Synthetic Biology/Engineering Biological Systems, Embrapa Genetic Resources and Biotechnology, Brasília 70770-917, Brazil; †† Institute of Biomedical Sciences, Department of Microbiology, University of São Paulo (USP), São Paulo, São Paulo 05508-000, Brazil

## Abstract

In the past two decades, synthetic biology has emerged as a transformative field at the frontier of innovation, evolving from fundamental technological advancements to the commercialization of innovative products. The field is providing breakthrough innovations to address societal challenges and has become a key technology for the sustainable development of global economies. This ongoing revolution represents an opportunity for countries to mitigate the damage caused by decades of reckless use of global resources. In Brazil, a growing community is harnessing this potential, driven by a shared vision and collaborative efforts. The establishment of the Brazilian Synthetic Biology Network marks a pivotal moment in bringing together diverse research groups and fostering interdisciplinary partnerships. By leveraging the nation’s rich biodiversity, scientific expertise, and bioproduction capacity, Brazil is well-positioned to lead advancements in sustainable solutions based on synthetic biology. To realize its potential, the country needs a strong and well-connected scientific community, long-term investments in infrastructure, and the development of domestic infrastructure for supplying critical materials, reagents, and core services that support synthetic biology research and innovation. The Brazilian Synthetic Biology Network represents a significant milestone in advancing Brazil’s biotechnological capabilities. While the Brazilian potential is clear for the Network members, the scientific and regulatory scenario in Brazil is poorly known by the international community. This review discusses key components of the synthetic biology ecosystem in Brazil and highlights the limitations that hinder the country’s technological development.

## Graphical abstract

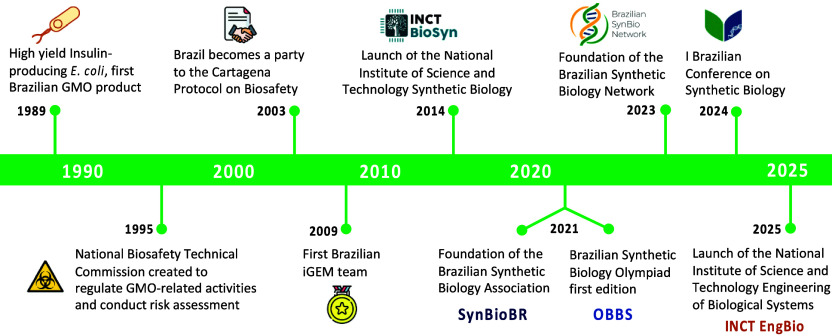

## Introduction

The world has witnessed an intense development of synthetic biology (SynBio) in the last two decades, which went from basic technological development to commercial products. The foundational idea that biology can become technology has instigated the development of many molecular tools to engineer cellular and cell-free systems and bring new solutions for societal challenges.^1–3^

Synthetic biology’s multidisciplinary and collaborative nature has shaped the formation of an enthusiastic community worldwide. The International Genetically Engineered Machine Competition (iGEM) helped to rapidly spread the word through universities and laboratories on all continents. Moreover, repositories such as the Registry of Standard Biological Parts (iGEM Foundation) and Addgene, and the Free Genes Project, promoted the sharing of biological parts that helped to establish new synthetic biology research groups around the world. However, the speed of spread was very different among regions. The United States pioneered synthetic biology development in the early 2000s, and the start of the Synthetic Biology Research Center (SYNBERC) in 2006 laid the foundations for the field in the USA. A few European countries such as The Netherlands, United Kingdom, and Germany joined the efforts with a short delay after 2004, with a field consolidation starting in 2010 after great initiatives such as massive funding for consortia in synthetic biology in The Netherlands,^4^ the publication of the UK Synthetic Biology Roadmap,^5^ and the establishment of the Max Planck Research Network in Synthetic Biology in Germany.^6^ It took a longer gap for synthetic biology to reach places such as Indonesia^7^ and African countries,^8^ with initiatives starting in 2014 and 2018 and still looking for a route to consolidate the field.

As in many other places, in South American countries, synthetic biology dissemination started with graduate and undergraduate students participating in the iGEM competition. A Colombian team was the first South American team to participate in the competition in 2006. The first Brazilian participation came in 2009. After that, Brazil experienced a lag phase of growth in synthetic biology, only experiencing exponential growth after 2014, starting with the creation of the National Institute of Science and Technology in Synthetic Biology (INCT BioSyn) and funding efforts from national grant agencies. Additionally, after 2014, iGEM teams formed in different parts of Brazil, helping to spread synthetic biology research throughout the country. Despite the active engagement of numerous universities and corporate laboratories in synthetic biology and related fields such as metabolic engineering and systems biology, a cohesive Brazilian community had not been successfully established until recently. Efforts to establish an organized community started in 2021 with the foundation of the Brazilian Association of Synthetic Biology (SynBioBR, discussed in detail in the next section), led by graduate students. Later in 2023, SynBioBR partnered with the INCT BioSyn to organize the Brazilian SynBio Forum, where 12 Brazilian research groups founded the Brazilian Synthetic Biology Network.

## The Brazilian Association of Synthetic Biology

SynBioBR was established in 2021, receiving its official status as a registered nonprofit entity in 2023. SynBioBR cultivates a community of students, academics, researchers, entrepreneurs, and synthetic biology enthusiasts across Brazil. Serving as a nexus, the association facilitates connections and collaborations, and offers specialized support for professionals in synthetic biology. By bridging diverse sectors, discerning specific challenges, and facilitating open discourse, SynBioBR cultivates an ecosystem that identifies opportunities and upholds equitable representation among stakeholders.

SynBioBR’s educational initiatives have demonstrated significant reach and impact. The First Brazilian Symposium on Synthetic Biology in 2021 attracted over 1500 online participants from all 27 Brazilian states. SynBioBR further underscored the significant impact of iGEM teams on Brazil’s synthetic biology scenario by organizing a satellite event for the 2021 iGEM competition.

Year-long iGEM projects, led by exceptional students and researchers, often yielded groundbreaking ideas. Recognizing this value, SynBioBR has actively committed to supporting their advancements. Beginning in 2024, the association took the lead in organizing the Brazilian Synthetic Biology Olympiad, first conceived by the team iGEM-USP-Brazil in 2021. The Olympiad engages over 3500 high school students annually to educate and foster an early interest in synthetic biology.

Central to SynBioBR’s mission is enhancing visibility of Brazil’s synthetic biology stakeholders. The association launched a newsletter with over 1600 subscribers, ensuring the community stays informed about current developments, events, and new publications from Brazilian groups. SynBioBR further fosters engagement through regular webinars showcasing individual, group, and startup research. A major milestone in community mapping came with the launch of the Observatory, an interactive map that centralizes information on research groups active in the field, offering a comprehensive view of the Brazilian SynBio community.^9^ The database will soon include startups and companies promoting synthetic biology nationwide.

SynBioBR’s growing influence in policy circles was recently recognized by the Brazilian Ministry of Science, Technology, and Innovation through an invitation to contribute to the national position on synthetic biology under the framework of the Convention on Biological Diversity.

In a strategic partnership, SynBioBR collaborated with the INCT BioSyn to establish the Brazilian Synthetic Biology Network. While the association takes a technical role, representing the community in policy discussions, organizing events, issuing technical opinions, and fostering the strategic development of synthetic biology at both national and international levels, the Network provides a more research-oriented branch. Together, these initiatives contribute to a long-term vision: propelling Brazil to the forefront of synthetic biology research. By empowering researchers, facilitating collaborations, and promoting knowledge exchange, they cultivate a dynamic landscape ripe for future breakthroughs.

## The Brazilian Synthetic Biology Network

Unlike the rapid expansion of synthetic biology in the United States and the current trends in Europe and China, Brazil has not experienced a comparable acceleration in either academic or industrial contexts. Nevertheless, there has been a notable increase in the number of groups engaged in this field across all regions of Brazil in recent years. However, due to the vast size of the country, these groups are not well-acquainted and encounter challenges in establishing connections and building collaborations. In this context, the Brazilian Synthetic Biology Network was founded to establish an organized community to strengthen and expand research and development in the field, influence political decisions on funding and biosecurity, and promote outreach and educational activities.

Formed in November 2023 and officially launched in August 2024, during the first Brazilian Conference on Synthetic Biology (I CBBS), the Network is growing fast, consisting now of 32 research groups from 18 Brazilian institutions located in all five regions of Brazil (Figure 1). The Brazilian Synthetic Biology Network currently has 66 affiliated researchers (PIs, postgraduates, graduates, and undergraduate students) working on synthetic biology projects.

**Figure 1:**
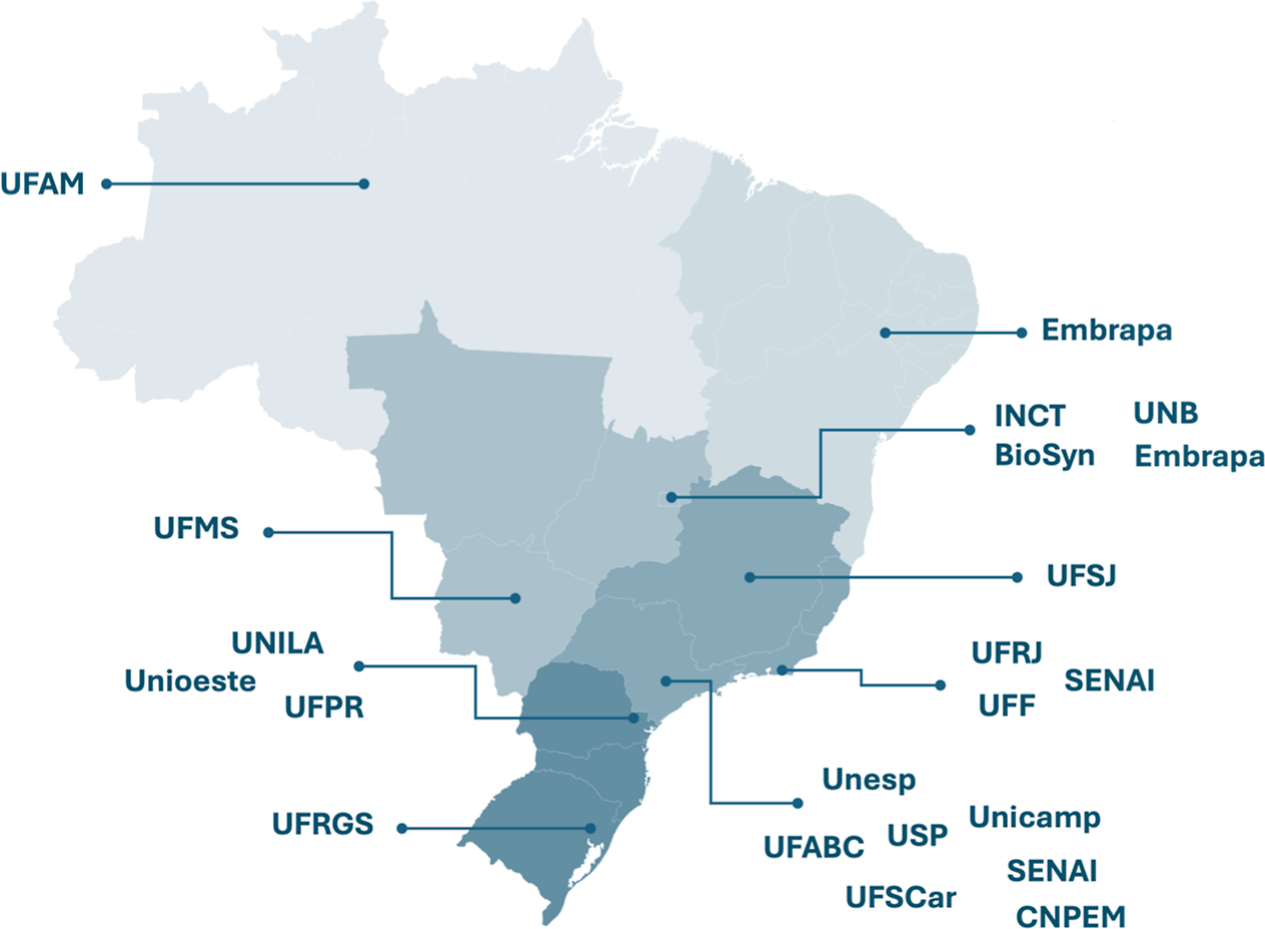
Brazilian Synthetic Biology Network brings together research groups from 18 institutions spread over all five Brazilian regions. Most of the research groups are located in the highly populated Southeast region, in public universities such as the São Paulo State University (UNESP), University of São Paulo (USP), State University of Campinas (Unicamp), Federal University of ABC (UFABC), Federal University of São Carlos (UFSCar), Federal University of Rio de Janeiro (UFRJ), Fluminense Federal University (UFF), and Federal University of São João Del-Rei (UFSJ) as well as innovation centers, two from the National Industrial Apprenticeship Service (SENAI), SENAI Biotechnology (ISI Biotecnologia) and SENAI CETIQT Biosynthetics and Fibers (ISI B&F), and the Brazilian Center for Research in Energy and Materials (CNPEM). The Brazilian Agricultural Research Corporation (Embrapa) has two research groups affiliated with the Brazilian Synthetic Biology Network, one in Petrolina in the Northeast region and another in the country’s capital Brasília. Brasília is also the home of the INCT BioSyn and the affiliated groups from the University of Brasilia (UnB). The fourth affiliated institution from the Midwest is the Federal University of Mato Grosso do Sul (UFMS). The other four affiliated groups are located in the Southern region, at the Federal University for Latin American Integration (UNILA), the Federal University of Paraná (UFPR), the Western Paraná State University (Unioeste), and the Federal University of Rio Grande do Sul (UFRGS). Finally, located in the heart of the Amazon, the Federal University of Amazonia (UFAM) is the affiliated in the Northern region.

Working side by side with SynBioBR, the Network has yielded high-impact projects, such as the Brazilian Biological Parts Repository. This online resource empowers researchers to share and access vital components like sequences, plasmids, and strains. Notably, the project was developed with consultations from the renowned organization, Asimov, further solidifying the repository’s adherence to best practices. Another fruitful collaboration resulted in the first Brazilian Conference on Synthetic Biology (I CBBS), which gathered researchers and professionals nationwide in August 2024 for a fruitful discussion on the latest advancements in the field. A second edition of the CBBS is scheduled for 2025 increasing the capacity from 80 to 160 attendees, a 100% expansion from the inaugural event. Ultimately, network members secured funding for the establishment of the National Institute of Science and Technology in Engineering Biological Systems (INCT EngBio). The INCT EngBio represents a natural evolution of the pioneering effort, the INCT BioSyn, to synergistically integrate biotechnology, biodiversity, artificial intelligence, and quantum computing to develop solutions aimed at addressing healthcare, bioproduction, and environmental challenges.

The Brazilian synthetic biology research landscape has diverse roots. Prior to our community-building efforts, some researchers did not explicitly identify their work as synthetic biology. However, despite variations in terminology and approaches, we share a common vision of engineering biological systems for technological applications. This shared aspiration serves as the foundation for our efforts to establish a unified Brazilian synthetic biology community. As a community, we strive to harness Brazil’s natural potential for biotechnology and synthetic biology through collaborations and sharing, and to engage future generations of scientists in the effort to convert biodiversity into technology.

## Brazil’s Potential in Synthetic Biology: Challenges and Strategic Imperatives

Brazil is a green giant that spans 8,515,759 km^2^ and stands out as the World’s most biologically diverse country. This vast richness includes six terrestrial biomes and three large marine ecosystems, harboring over 125,000 animal species and 50,000 plant species, accounting for 70% of the cataloged global biodiversity. With an estimated 15–20% of the Earth’s total biological diversity, Brazil stands out as the nation with the highest number of endemic species globally.^10,11^ Managing this vast biodiversity presents both a scientific challenge and an opportunity to contribute to sustainable development.

Brazil’s vast biodiversity uniquely positions the nation to spearhead advancements in biotechnology and synthetic biology, particularly across the healthcare, agriculture, and environmental sustainability sectors (Figure 2). Country’s diverse natural ecosystems are a rich reservoir of unique bioactive compounds, many of which are currently under investigation for their therapeutic potential. Synthetic biology offers a sustainable pathway for replicating and mass-producing these natural molecules using engineered biological systems, thereby presenting a viable alternative to direct extraction from native species. In the agricultural domain, synthetic biology can leverage genetic information from native Brazilian species to develop crops with enhanced resilience and productivity. Furthermore, the country’s ecosystems not only provide valuable genetic resources but also serve as crucial platforms for testing and implementing synthetic biology-based environmental solutions in real-world scenarios. This approach holds particular significance given the international frameworks of the Convention on Biological Diversity and the Nagoya Protocol, which govern access to genetic resources and advocate for fair and equitable benefit-sharing. In Brazil, a Federal Decree officially enacts the Nagoya Protocol, underscoring the agreement’s objective: to ensure the equitable sharing of benefits derived from the utilization of genetic resources through appropriate access, technology transfer, and adequate funding. This framework directly contributes to both biodiversity conservation and the sustainable use of its components. By embracing this dual role—as both an inspiration for biological innovation and a setting for responsible technological deployment—Brazil is well-positioned to make substantial contributions to global challenges, all while safeguarding its biodiversity through sovereign and sustainable innovation.

**Figure 2:**
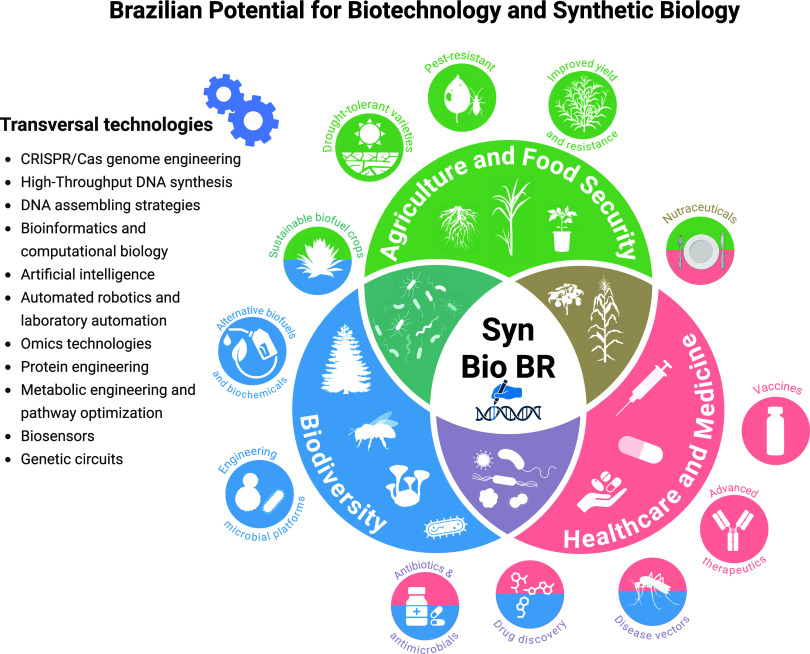
Brazilian potential for biotechnology and synthetic biology. The infographic illustrates Brazil’s vast potential in synthetic biology and biotechnology, highlighting key applications in three crucial areas: **Healthcare and Medicine**, where the country’s rich biodiversity offers opportunities for innovative therapies and drug development; **Agriculture and Food Security**, showcasing advancements in crop yield enhancement, pest resistance, and sustainable farming practices; and **Environmental Sustainability and Biodiversity**, emphasizing biotechnological strategies to preserve ecosystems and protect the nation’s unparalleled biodiversity. Together, these examples demonstrate how Brazil’s natural resources and scientific advancements position it as a leader in addressing global challenges in these sectors.

One notable approach for harnessing biodiversity is the synthetic domestication of valuable traits.^12^ For example, the MaSp2-like gene from the Amazonian spider *Avicularia juruensis* was harnessed for the production of recombinant spider silk proteins. The gene features unique motifs such as (GA)­n, poly-A, and GPGXX, which are crucial for the exceptional strength and durability of spider silk. By integrating these motifs into bacterial or other expression systems, researchers can produce large quantities of synthetic spider silk for a range of applications.^13^ While this exploration of biodiversity underscores Brazil’s potential for scientific advancement, the country’s extensive natural resources also pave the way for significant contributions to practical biotechnological solutions. However, realizing this potential requires navigating the complex relationship between biodiversity and synthetic biology, which presents both opportunities and challenges. On one hand, biodiversity provides a source of inspiration and resources for bioprospecting genes and organisms that can be explored to discover new biological functions and biomolecules. On the other hand, synthetic biology offers new tools for exploring and conserving biodiversity. This dual nature necessitates a framework that recognizes Brazil’s biodiversity as both a source of innovation and a critical resource requiring protection.

Indeed, the country has some of the strictest environmental laws on the planet. The rigidity of Brazilian environmental legislation reflects the complexity and importance of its natural heritage, as well as the need to balance economic development with environmental conservation. Thus, in Brazil, in legal terms, strict environmental standards are seen as essential for ensuring the protection of ecosystems and the well-being of present and future generations.

Brazil is a signatory to several international agreements and conventions on the environment, such as the Convention on Biological Diversity and the Paris Agreement. These commitments also drive the country to maintain high standards of environmental protection. Based on these conventions, Brazil formulated the Biodiversity Law, which regulates access to genetic heritage and associated traditional knowledge. Genetic heritage refers to all the information concerning the genetic origins of organisms. Associated traditional knowledge is the collection of knowledge acquired by indigenous populations, traditional communities, and family farmers about the direct or indirect use associated with genetic heritage. Researchers are required to register their activities on the SisGen (Genetic Heritage Management System and Associated Traditional Knowledge) platform if they involve access to or research with the Brazilian biodiversity. By definition, access to biodiversity refers to applying or economically exploiting the Brazilian genetic heritage and associated traditional knowledge, while research refers to experimental or theoretical activities performed using Brazil’s genetic heritage and associated traditional knowledge to produce new scholarship. Foreign researchers are required to partner with a Brazilian research institution to access or research Brazilian biodiversity. Actions within the scope of the Biodiversity Law are regulated by the Genetic Heritage Management Council (CGen) linked to the Ministry of the Environment and Climate Change. These regulatory frameworks ensure responsible stewardship of national genetic resources and operate alongside the country’s established strengths in biotechnological production.

With large arable lands and a favorable climate, Brazil stands out too as one of the largest producers of agricultural commodities. The confluence of Brazil’s expansive land area, rich biodiversity, and suitable climatic conditions presents great prospects for the advancement of biotechnological development. Bioethanol can be produced from a wide variety of renewable feedstocks, and the choice of feedstock significantly impacts production costs. Sugar cane, a C-4 photosynthetic species, exhibits exceptionally high biomass productivity.^14^ As a result, Brazil is the world’s leading producer, consumer, and exporter of sugar cane ethanol, accounting for 28% of the world’s total production as of 2023. The technology for ethanol production from sugar cane, developed in the 70s and fully implemented in the 80s, reduced the national dependence on imported oil, strengthened the economy, and diversified the country’s renewable energy portfolio. Brazil’s leading status in the bioethanol sector is also attributable to the substantial investments in discovering and developing new engineered *Saccharomyces cerevisiae* strains for enhanced ethanol production. Over the past decades, researchers have conducted extensive efforts to isolate, characterize, and integrate a variety of strains into production processes, thereby advancing our scientific understanding of ethanol-fermenting yeasts. These strains have served as valuable platforms for subsequent engineering aimed at producing bioethanol from lignocellulose-derived sugars. One engineered strain, capable of converting xylose into second-generation ethanol at a commercial scale,^15^ is currently employed in Granbio’s biorefinery. These achievements exemplify how coordinated efforts between scientists, government, and industry can drive significant technological advancement.

In parallel, Brazil has pioneered advancements in sugar cane cultivation. The country approved the world’s first engineered sugar cane in 2017. Developed by the Sugar Cane Technology Center (CTC), the transgenic variety is less susceptible to cane borer infestation.^16^ With safety approvals secured in major markets, including the United States and Canada, Bt Cane and its derived sugar products have gained global recognition. To further accelerate innovation, CTC has partnered with Ginkgo Bioworks to develop novel pest control molecules combining CTC’s agronomic expertise with Ginkgo’s engineering platform. Ginkgo Bioworks has also established a strategic partnership with Agrivalle, a Brazilian agricultural biological inputs company. This collaboration aims to enhance the portfolio of biocontrol products, including bioinsecticides, biofertilizers, and biofungicides, through microbial strain optimization.

In addition to sugar cane, Brazil has successfully developed transgenic cultivars of other key crops, including soybeans, cotton, and corn. The Brazilian Agricultural Research Corporation (Embrapa) has played a pivotal role in these advancements, with notable examples including glyphosate-resistant cotton varieties (cultivar BRS 370 RF) and corn cultivars engineered to resist both caterpillars and herbicides (cultivar BRS 3042 VT PRO2). Embrapa has also developed transgenic beans resistant to the golden mosaic virus, a major threat to Brazilian bean production, using RNA interference technology (cultivar BRS FC401 RMD). Another example is the enhancement of soybean oil profiles through the genetic study of fatty acid metabolism in oilseed plant species native to Brazil, giving higher oleic acid and lower palmitic acid contents. This increases their suitability for biofuel production and similar industrial uses.^17^ The developments made by Embrapa contribute to more sustainable agricultural practices, aligning with global efforts to reduce the environmental impact of farming while ensuring food security.

Brazil’s contributions to biotechnology extend beyond agriculture, with significant advancements in health. A notable example is the development of recombinant insulin, the nation’s first genetically engineered product. In 1988, *Biobrás Bioquímica do Brasil* partnered with Prof. Dr. Spartaco Astolfi-Filho from the University of Brasilia to develop a recombinant insulin alternative to the animal-derived insulin traditionally extracted from pig pancreas.^18^ The initiative resulted in a high-yielding insulin-producing *Escherichia coli* strain. Biobrás initiated recombinant insulin production and testing in late 1989, securing patents in the USA and Brazil in 2000 and 2012, respectively. Although Biobrás was acquired by Novo Nordisk in 2001, its legacy continues through Biomm S.A., a spinoff company that resumed insulin production in Brazil in 2024, albeit with a different technological approach.

Brazil has witnessed a significant surge in biotechnological innovation in the pharmaceutical sector as well. A prime example is Cristália, a pharmaceutical company that has invested heavily in developing biological products derived from engineered bacterial strains. Under the guidance of Prof. Dr. Astolfi-Filho, Cristália established its biotechnology sector in 2006. A notable achievement was the development of a recombinant human growth hormone (somatropin) biosimilar. The engineering technology for the bacterial strain was developed at the Federal University of Amazonas (UFAM) and transferred to Cristália.^19^

Building upon the trend of innovative biotechnological developments, Brazil is also exploring the potential of personalized therapies. The Brazilian National Cancer Institute (INCA) and the Oswaldo Cruz Foundation (FIOCRUZ) operate a collaborative research program dedicated to developing new technologies based on CAR-T cells.^20^ The innovative approach aligns with a point-of-care strategy, with the potential to increase the accessibility and rate of CAR-T therapy delivery. Additionally, INCA is actively involved in initiatives to integrate well-established protocols of CAR-T therapy into Brazil’s public healthcare network.

Brazil’s biodiversity richness provides essential ecosystem services fundamental for the Earth’s sustainability, such as climate regulation, water conservation, and pollination, which are fundamental for the sustainability of the planet. Consequently, Brazil must have strict environmental laws to protect its biodiversity and prevent biopiracy.

## Biosafety and Biosecurity

As one of the world’s largest producers and exporters of agricultural commodities, genetic engineering to improve the natural features of crops has guaranteed the favorable positioning of the agro-industry sector in the country’s trade balance over the decades.^21^

Since the emergence of cloning technologies in the 1970s, it has become evident that organisms could be genetically enhanced, and recent breakthroughs and discoveries in genome editing technologies have further expanded the scope of possibilities for designing organisms with superior traits. Currently, of the plant crops cultivated in Brazil, 92% of soybeans, 90% of corn, and 47% of cotton are genetically engineered.^22^ Beyond crop plants, over the past decades, various microorganisms, enzymes, insects, and vaccines for animals and humans have been developed in Brazil using diverse engineering strategies, fostering the need for precautionary biosafety regulations to assess and manage potential risks.

The first commercially genetically engineered crop produced in the world was the FLAVR-SAVR tomato in 1994, modified to delay ripening and improve shelf life in the postharvest period. After this breakthrough, discussions about biosafety and the creation of regulatory systems to monitor and control the production and distribution of GMOs began to take place around the world. The discussion started in Brazil in the early 90s and resulted in the approval of the first Legal Biosafety Regulatory System in 1995, which set the rules for the development, cultivation, manipulation, transportation, marketing, consumption, release, and disposal of GMOs.^23^ In the same year, the National Biosafety Technical Commission (CTNBio) was created to institute biosafety policies and regulations governing activities and projects involving GMOs, operating under the Ministry of Science, Technology, and Innovation. CTNBio comprises experts from various disciplines, conducts rigorous risk assessments to assess the safety and efficacy of GMOs, and is responsible for evaluating and approving GMOs for environmental release and commercialization.^21^

Besides CTNBio, the Brazilian GMO regulatory system is also represented by the National Biosafety Council (CNBS), Internal Biosafety Committees (CIBios), and the Surveillance and Registration Bodies.^22,23^ The process of approval and commercial release of GMOs in Brazil starts with the CIBio of a public or private institution providing CTNBio with a comprehensive GMO risk assessment study. This study encompasses detailed information concerning GMO risk classification, including specifics regarding the engineered genes, such as their function, origin, and engineering methods. The proposal must also address aspects such as spore production and desiccation resistance, agents with sterilizing activity against the GMO, potential impacts on water, air, and soil quality, as well as the organism’s ability to survive and disperse in the environment. Additionally, for GMOs intended as food, a thorough assessment of risks to human and animal health must be conducted.^24^ While CTNBio assesses risks and authorizes commercial approval, the CNBS evaluates the potential negative impacts of new products on social and economic levels and establishes the National Biosafety Policy.^22^

So far, CTNBio has issued hundreds of approvals for the commercial release of engineered vaccines, crops, insects, and microorganisms in Brazil. However, with these advancements come significant ethical and safety considerations. As the application of synthetic biology continues to expand, it becomes imperative to establish robust biosafety and biosecurity frameworks to mitigate potential risks associated with the release of GMOs into the environment.

In the international arena, Brazil has long cooperated with efforts to implement biosafety and biosecurity frameworks. The country was one of the original 38 signatories of the 1925 Geneva Protocol, which prohibits the use of chemical and biological weapons in war. Brazil also ratified the 1972 Biological Weapons Convention, the first multilateral disarmament treaty to ban the production of an entire category of weapons of mass destruction. In 2003, Brazil became a party of the Cartagena Protocol, recognizing the importance of biosafety regulation in the context of biotechnology.

Domestically, biological agents with potential risk for use in bioterrorism have been regulated as sensitive goods since 1995, and the Anti-Terrorism Law from 2016 defined terrorism as the use or threat of use of biological materials capable of causing harm or promoting mass destruction.

## Students as Catalysts of the Brazilian Synthetic Biology Community

Undergraduate and graduate students at Brazilian universities played a pivotal role in the early development of synthetic biology in the country. Organized as teams for the iGEM competition, they spread the word throughout the universities and helped to gather interest in the area. Brazil’s first participation in iGEM took place in 2009 with the UNICAMP-Brazil team. Since then, there has been a significant increase in Brazilian participation. The consistent participation of multiple teams from USP (USP-Brazil, USP-EEL-Brazil, and USP-São Carlos-Brazil), along with the UFMG-Brazil and UFAM-Brazil teams, has strengthened Brazil’s presence in the iGEM competition. Building upon early medal achievements, Brazilian teams began securing prestigious special prizes in 2015 with UFMG-Brazil, and in 2018 with UNESP-Brazil and USP-Brazil. Notably, the USP-Brazil team reached the top-10 ranking in the 2021 competition. This sustained success has been accompanied by a significant rise in Brazilian participation. As of 2024, 15 different Brazilian teams have presented a total of 40 projects at the iGEM competition.

In the competition context, Brazilian iGEM teams have integrated synthetic biology into social, academic, and entrepreneurial spheres. By addressing local problems, they’ve fostered community dialogue and partnered with companies and research institutions. These partnerships facilitate technology transfer and support startup creation. Within the academic scope, the skills and experiences acquired during the iGEM competition are highly valued, providing a solid foundation for advanced research and professional development. After the initial participation in iGEM, many undergraduates seek to perpetuate their learning by establishing synthetic biology clubs. Formed mostly by undergrad students interested in delving into complex synthetic biology topics, their activities provide students with a platform to learn and apply synthetic biology concepts through hands-on projects. In addition to improving technical skills, the clubs encourage collaborations between students from different areas, expanding their understanding and ability to integrate knowledge, and improving their soft skills. Moreover, members of the clubs build a network of contacts that can benefit their future careers.^25^ Going beyond, UNILA-LatAm’s SynFronteras Club extended its reach to high schools, inspiring and supporting the creation of Brazil’s first high school iGEM team in 2023.

The collective efforts of Brazilian iGEM teams have significantly contributed to the advancement of synthetic biology within the country. Through their active participation in the iGEM competition and their subsequent engagement in various initiatives, the teams have not only enhanced their own skills and knowledge but have also played a pivotal role in fostering the growth of the synthetic biology community. The establishment of educational programs by Brazilian iGEM teams such as the UFMG’s Biological Machine Engineering Summer School, with over six editions completed, and USP’s Brazilian SynBio Olympiad, demonstrates the lasting impact of iGEM on the cultivation of future generations of synthetic biology researchers and innovators in Brazil.

## Synthetic Biology in the Brazilian Academia

Since its inception in the early 2000s, synthetic biology has rapidly advanced, prompting professionals in various branches of biology, engineering, and chemistry to continuously update their knowledge and skills to keep pace with the latest developments. The dynamic and interdisciplinary nature of the field requires that educators create and maintain cutting-edge courses to prepare the next generation of innovators and researchers.

In that context, 16 Brazilian universities offer synthetic biology courses at the undergrad and graduate levels as a key curriculum component for future professionals, equipping them with the knowledge and skills to tackle complex biological challenges and drive innovation in diverse scientific and industrial applications. In comparison, as of 2021, Canada had only three universities offering synthetic biology courses.^26^ The Brazilian courses are spread over all five country regions, and, given Brazil’s vast continental size, this extensive distribution of knowledge in synthetic biology is important for fostering innovation and development among Brazilian students and society.

The dissemination of synthetic biology in Brazil is steadily expanding, driven by initiatives within universities and research institutions. These efforts foster a growing interest and understanding of this innovative field among students, researchers, and professionals nationwide. With synthetic biology clubs, educational courses, and research projects becoming increasingly prevalent, the Synthetic Biology Brazilian Network is positioned to contribute to advancements in biotechnology and sustainable development, underscoring its commitment to shaping the future of synthetic biology nationally and globally.

## Synthetic Biology-Related Entrepreneurship and Industry in Brazil

Over the past decade, Brazil has experienced significant advancements in synthetic biology and biotechnology, with established companies and start-ups emerging as key players in these fields. The majority of these companies commercialize products and services related to human health and well-being, agriculture, commodities, and animal health. Braskem, a Brazilian petrochemical company that profits from petrochemicals, invests in synthetic biology research and development and is a leader in the production of thermoplastic resins from renewable sources (polyethylene, polypropylene, and polyvinyl chloride) in the Americas. Additionally, large fuel companies such as GranBio and Raízen are investing in synthetic biology to develop modified microbial strains for their biorefineries processing sugar cane as a raw material. Although synthetic biology is still in its early stages in Brazil, the field is witnessing a growing number of startups emerging. Notable examples are BioLinker, a young company that offers a diverse portfolio of synthetic and molecular biology products and services; Vyro Biotherapeutics, develops treatments for central nervous system tumors using a genetically engineered Zika virus to target and kill tumor cells without harming neurons or other healthy cells; InEdita Bio, works to enhance crop efficiency and disease resistance through gene editing; GALY & CO., grows cotton from plant cells in bioreactors; GEN-T, works to boost precision medicine through genome sequencing; MUSH, uses agro-industrial waste to create durable and biodegradable materials.

Brazil ranks as the ninth country in terms of the number of biotechs founded, with approximately 350 active biotechs in 2023. In Latin America, Brazil accounts for 60% of biotechs. However, biotechs struggle to survive, with failure rates estimated at 95% in their first years.^27^ Even when they do survive, large international companies work to stifle them and avoid competition. For example, the Brazilian companies Alellyx and CanaVialis, specialized in the development of genetically engineered crops, were sold to Monsanto (later acquired by Bayer) in 2008. Both Brazilian companies had been heavily funded with public money. Seven years later, Monsanto shut down the activities of both Brazilian subsidiaries.

Brazil’s biotech industry is largely composed of young companies, the majority less than 5 years old and typically small. The industry benefits significantly from robust connections with universities and research centers, which are crucial to its growth. These companies usually operate with lean teams, averaging around 20 employees, yet they boast a highly educated workforce. Notably, among companies with 1–10 employees, 40% of professionals hold PhDs, and approximately 20% possess MSc degrees. While 25% of these companies export their products, a substantial 86% rely on imports, mainly for reagents and equipment. Additionally, 78% of companies receive support from governmental agencies such as the Studies and Projects Financing Agency (FINEP), the National Council for Scientific and Technological Development (CNPq), the National Bank for Economic and Social Development (BNDES), as well as state foundations. Despite these advantages, venture capital remains underutilized, with only 14% of companies having secured such funding. One of the key funding programs supporting the creation and development of startups is the Innovative Research in Small Businesses Program (PIPE) from the São Paulo Research Foundation (FAPESP). The PIPE program fosters innovation and supports research in small and medium-sized enterprises (SMEs) throughout the São Paulo State, Brazil, providing essential funding and resources to assist businesses in developing new technologies, products, and processes. By bridging the gap between scientific research and practical application, PIPE-FAPESP encourages collaborations between researchers and industry professionals. In doing so, it significantly contributes to the growth of the local economy and enhances the competitiveness of São Paulo’s biotech sector.

## Synthetic Biology Worldwide: Regional Dynamics and Prospects

Brazil invests 1.2% of its gross domestic product (GDP) in research and development (Agência Brasil, 2024), the highest share among Latin American countries, lagging just behind countries such as Italy (1.3%) and Australia (1.6%), and way behind leaders such as China (2.6%), the USA (3.4%), and South Korea (4.96%). Brazil is a leader in Latin America in the number of published papers in the areas of synthetic biology and metabolic engineering, with 480 papers, followed by Chile with 165. As a comparison, Italy and Australia have 1058 and 953, respectively. The leaders, the USA and China, have published over 10,000 papers each (Figure 3A). From all published papers by Brazilian-affiliated scientists in these areas, 42% have been published in the last 5 years (Figure 3B), showcasing a growth in recent years. The most impressive recent growth comes from China, with 55% of all papers published in the last 5 years.

**Figure 3:**
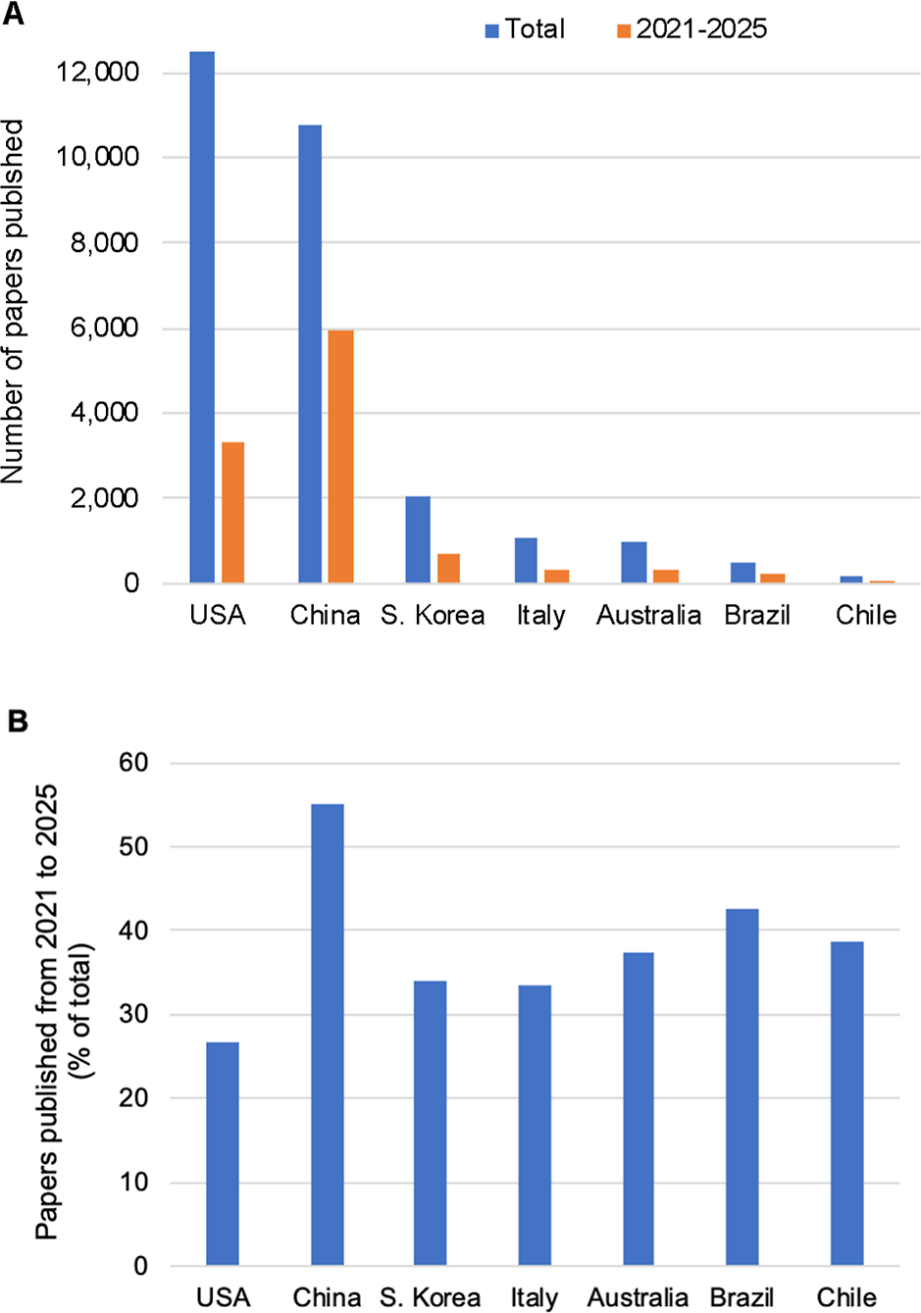
Academic output as papers published by countries in synthetic biology. (A) Number of papers published all time. (B) Papers published in the last 5 years (2021–2025) as a percentage of the total. Search was carried out on Scopus using the country name for affiliation AND “synthetic biology” OR “metabolic engineering” as keywords.

On a bigger scenario, Brazil is the fifth country with the highest number of publications in biological and agricultural sciences,^27^ and the leader in Latin America in scientific publications and citations, as evidenced by international indices such as the Nature Index and Scimago Journal & Country Rank. However, this scientific production has still not been converted into business generation. The Brazilian government has recently announced efforts to support innovation and business generation, such as the goal to reduce the time for granting patents from 4 to 2 years, and for trademark registration from 18 months to just 1 month by 2026.

The world’s synthetic biology market reached 16.35 billion USD in 2024 and it has been estimated to grow at a Compound Annual Growth Rate (CAGR) of 17.31% from 2025 to 2034. The global synthetic biology market is characterized by distinct regional dynamics. North America, led by the USA, commands 40% of this billionaire market. This is a fast-growing market, substantially driven by millionaire annual US federal research and development funding and robust private investment.^28^ The region’s ecosystem is further distinguished by a strong venture-backed commercialization model and a comparatively flexible regulatory environment. The European market grows supported by organizations such as the European Synthetic Biology Society (EUSynBioS). Europe combines exceptional academic output, measured as the number of papers published, and robust sustainability policy. However, despite its globally competitive academic research, Europe faces challenges in translating fundamental discoveries into applied technologies, as evidenced by a significantly lower number of patents granted compared to the USA.^3^ The Asia-Pacific market is experiencing the most rapid growth at a CAGR of 22.14%. Notably, China has surpassed Europe in producing high-impact biotechnology papers and patents, a development supported by heavy and sustained investments.^28^

Although still very modest, the Brazilian synthetic biology market has been growing steadily in recent years. In 2023, the market was valued at approximately 214.6 million USD, and it is projected to grow at a CAGR of 21.2% until 2030.^29^ One landmark for the recent development of synthetic biology in Brazil was the 2012 event “Bio-economy: Developing an Agenda for Brazil”, organized by the National Confederation of Industry (CNI) in partnership with the Harvard Business Review Brazil to propose a Bioeconomy agenda for Brazil for the decade 2013–2022. The event highlighted the need to increase government investments in the rehabilitation, upgrade, and expansion of the national infrastructure to advance research and qualify human resources in strategic fields such as synthetic biology, genomics, proteomics, and biomaterials. Since then, the financial support for these fields has increased significantly.

The advancement of a sustainable and innovation-driven bioeconomy in Brazil has garnered significant attention with the establishment of a National Comission on Bioeconomy (CNBio) in 2024. CNBio is a multistakeholder body tasked with the formulation and oversight of a National Plan for Bioeconomy Development (PNDBio). The PNDBio aims to promote the sustainable use of biodiversity and biomass, stimulate innovation ecosystems, support sociobiodiversity-based activities, and enhance Brazil’s competitiveness in global value chains. The initiative aligns bioeconomic development with low-carbon strategies and positions Brazil’s biodiversity as a strategic asset for technological and economic transformation.

At the multilateral level, Brazil’s 2024 presidency of the G20 marked a pivotal moment for international bioeconomy governance through the launch of the G20 High-Level Principles on the Bioeconomy. These 10 voluntary, nonbinding principles, endorsed by G20 member states, emphasize sustainability, equity, climate resilience, and the integration of traditional knowledge and modern science. Brazil’s bioeconomy vision mirrors many of the G20 priorities, reinforcing the country’s leadership in shaping a globally relevant, yet locally rooted, bioeconomy agenda.

## Concluding Remarks

Brazil holds great potential for synthetic biology, and the Brazilian Synthetic Biology Network is committed to accelerating the field’s progress. However, to fulfill its potential, the country needs to catch up with the world leaders in terms of funding and infrastructure.

Brazil is uniquely positioned to contribute to the global synthetic biology landscape, owing to its rich biodiversity and robust academic foundation. However, the country currently lacks the integrated funding frameworks, venture capital infrastructure, and biofoundries that characterize leading ecosystems in the USA, EU, and China. Brazil’s regulatory environment, particularly following the implementation of the Nagoya Protocol, provides a foundational basis for sovereign access to genetic resources and benefit-sharing. Nevertheless, a clearer alignment between regulatory frameworks and innovation incentives is necessary to catalyze commercialization pathways and facilitate the creation of spin-out companies.

To transition from its current academic leadership to global competitiveness in synthetic biology, Brazil must prioritize strategic investments. These investments should focus on developing critical infrastructure, including national-scale biofoundries and advanced synthetic biology platforms. Fostering public-private partnerships to stimulate venture-backed startups will also be crucial. One major bottleneck is the lack of companies producing high-quality equipment and chemicals locally to supply the laboratories, as well as essential services such as gene synthesis. Currently, Brazil relies on expensive and time-consuming imports, where products can take weeks to arrive due to distance and import regulations. Establishing local synthesis capabilities would dramatically increase access to synthetic DNA and speed up research projects. Additionally, research and development in Brazil rely heavily on an underpaid workforce maintained with poor scholarships, making it less attractive to talented students and hindering the development of innovative and cutting-edge technologies. Harmonizing legal frameworks with innovation ecosystems, securing dedicated funding (both external and domestic), and establishing robust talent pipelines through specialized synthetic biology training programs are essential steps. By implementing these measures, Brazil can leverage its distinctive bioresource advantage to foster domestic innovation and emerge as a prominent facilitator in the international synthetic biology panorama.
